# Periodontal and peri-implant bleeding on probing in patients undergoing supportive maintenance: a cross-sectional study

**DOI:** 10.1007/s00784-024-06030-5

**Published:** 2024-11-07

**Authors:** Antares Outatzis, Katrin Nickles, Hari Petsos, Peter Eickholz

**Affiliations:** Center for Dentistry and Oral Medicine (Carolinum), Dept. of Periodontology, Goethe-University Frankfurt, Theodor-Stern-Kai 7, 60596 Frankfurt am Main, Germany

**Keywords:** Dental implants, Periodontal probing, Peri-implant probing, Bleeding on probing

## Abstract

**Objective:**

Assessment of periodontal and peri-implant inflammation, evidenced by bleeding on probing (BOP), among partially dentate patients receiving supportive periodontal care (SPC).

**Material & methods:**

Patient charts from the Center for Dentistry and Oral Medicine of Goethe-University Frankfurt with at least one dental implant were reviewed. Measurements included probing pocket depth (PPD), BOP, and full-mouth bleeding and plaque scores for all teeth and implants.

**Results:**

100 patients (median; lower/upper quartile: age 68.9; 62.6/76.5 years; 51 females, 6 smokers, 16 with anticoagulative medication, 6 localized stage III, 57 generalized stage III, 37 stage IV, 70 grade B, 30 Grade C, 22; 20/25 teeth left, 2; 1/4 implants) were examined. Peri-implant BOP (24; 11.5/41.5%) was significantly higher than BOP at teeth (14; 8/21.5%) (*p* < 0.001). A median of 0 (0/1) implants exhibited no BOP and 0 (0/1) only one site with BOP. Shallow pockets (PPD 1–3 mm) were significantly more frequent in teeth (93; 87/97%) than in implants (72.5; 58/94.5%; *p* < 0.001). Moderately deep pockets (PPD 4 and 5 mm) were less frequent in teeth compared to implants (6; 2/11%; 22; 5.5/33%; *p* < 0.001).

**Conclusions:**

Peri-implant sites exhibit a higher prevalence of BOP compared to periodontal sites in SPC patients.

**Clinical relevance:**

Practitioners providing supportive periodontal care to patients with dental implants should anticipate a greater prevalence of BOP around implants compared to teeth.

## Introduction

Both periodontal and peri-implant bleeding on probing (BOP) induced by gentle probing using a periodontal probe are indicators for subgingival/submarginal inflammation [1–4]. Periodontal BOP (percentage of all bleeding sites per patient) and probing pocket depth (PPD) determine the case definition of gingival health, localized or generalized gingivitis (gingival health [BOP < 10%], localized [10–30%] or generalized [> 30%] gingival inflammation) [1], whereas peri-implant BOP determines the respective peri-implant parameters [2]. Several periodontal risk assessment scores (e.g., periodontal risk calculator, periodontal risk assessment) [5, 6] and the Implant Disease Risk Assessment (IDRA) [7] use the average BOP score per patient to categorized different levels of risk for periodontal/peri-implant disease.

In periodontal compromised but successfully treated patients under supportive periodontal care (SPC) PPD and BOP should be assessed at least once a year [8, 9]. In patients treated with dental implants PPD and BOP are assessed at implants as well [10, 11]. Merli et al. 2017 demonstrated that bleeding on peri-implant probing is significantly associated with PPD. However, they did not compare BOP between teeth and implants [12]. However, to distinguish periodontal and peri-implant health or disease within the same patient the BOP score should be assessed separately for teeth and implants. By doing so within the department of periodontology, we noticed in clinical practice that BOP score at teeth was consistently lower than at implants. Farina et al. 2017 took also PPD measurements at contralateral teeth into account and did not find a difference between teeth and implants. However, the cohort consisted of individuals seeking care at a research centre for the study of periodontal and peri-implant diseases and, thus, is likely to consist of untreated patients [13]. Based on our observation and the gap in the scientific literature we set up this cross-sectional study. This observation suggests a difference in clinical response to probing between teeth and implants and aligns with prior results conducted by the research group, which demonstrated a higher level of discomfort during peri-implant probing compared to periodontal probing [14–16]. Peri-implant mucositis (peri-implant BOP) is the precursor of peri-implantitis. Prevention and treatment of peri-implant mucositis prevent peri-implantitis [10]. If this actually is the case the hypothesis is expressed that higher frequencies of BOP at peri-implant sites compared to periodontal sites are an indicator that implants in patients with a history of periodontitis are more prone to develop peri-implantitis than periodontal compromised teeth to develop periodontitis.

To provide scientific evidence for the observation that the BOP score at teeth is consistently lower than at implants this retrospective cross-sectional study was planned in periodontal compromised but treated patients under SPC with at least one dental implant.

## Materials and methods

### Patients

This study was designed as retrospective cross-sectional study at the Department of Periodontology of the Center for Dentistry and Oral Medicine (Carolinum), Johann Wolfgang Goethe-University Frankfurt/Main. It complied with the rules of the Declaration of Helsinki and was approved by the Institutional Review Board for Human Studies of the Medical Faculty of the Goethe-University Frankfurt/Main (Application# 2021 − 372). The Study was registered June 5, 2023 at the German Registry of Clinical Studies (DRKS) under the ID DRKS00032005.

All charts of patients attending SPC at the Department of Periodontology of the Center for Dentistry and Oral Medicine (Carolinum), Johann Wolfgang Goethe-University Frankfurt/Main were screened for fulfilling the following inclusion criteria (October 2021 to September 2022).

#### Inclusion criteria

- At least 18 years of age.
- Participation in SPC at the Department of Periodontology of Goethe-University.
- Insertion of at least one dental implant.
- Comprehensive periodontal examination during SPC visit (modified Gingival Bleeding Index (GBI) [11] and plaque using the modified Plaque Control Record (PCR) [12], PPD, CAL-V, BOP) by one of two periodontally trained dentists (HP, PE).

#### Exclusion criteria

- Periodontal examination during SPC visit (PPD, CAL, BOP) by an undergraduate student.
- Missing radiographs.
- Violation of inclusion criteria.

Smoking status, and diabetes mellitus were retrieved from the charts. Patients who reported not to smoke were classified as non-smokers. Patients who reported smoking were classified as light (< 10 cigarettes/day) or heavy (≥ 10 cigarettes/day) smokers. Patients suffering from diabetes mellitus were classified as metabolically well (HbA1c < 7%; self-report) or uncontrolled (≥ 7%) [17]. Further, patients were asked for anticoagulative medication.

### Supportive periodontal/peri-implant therapy

At the Department of Periodontology of Goethe University SPC is rendered according to a standardized scheme [18]: Gingivitis is assessed using the GBI and plaque using the PCR at six sites per tooth/dental implant (mesiobuccal, buccal, distobuccal, mesiooral, oral, distooral). Patients received re-instruction and re-motivation for an effective individual plaque control and professional mechanical plaque removal (PMPR) with hand instruments and polishing by use of rotating rubber cups with polishing paste (SuperPolish, Kerr GmbH, Biberach, Germany) at teeth and by low abrasive air water abrasion (E.M.S., Electro Medical Systems S.A., Nyon, Switzerland) with glycine powder at implants (3 M Clinpro Glycine Prophy Powder, 3 M Germany GmbH, Neuss, Germany). After PMPR PPD and CAL were measured at 6 sites at each tooth and dental implant using a rigid steel probe (PCPUNC15, HuFriedy, Chicago, USA) to the nearest 1 mm. Thirty seconds after probing BOP was scored. In 9 patients PPD and CAL measurements in one quadrant were repeated by the respective other examiner to assess interexaminer reproducibility. Despite repeated measurements, these examinations were performed routinely as part of periodontal charting and were not performed for the purpose of the present study. At sites with PPD = 4 mm + BOP or PPD ≥ 5 mm subgingival instrumentation was rendered with curets [8] and 1% chlorhexidine digluconate gel (Chlorhexamed 1% gel, GlaxoSmithKline GmbH, Munich, Germany) was instilled. At PPD ≤ 5 mm and implants low abrasive air water abrasion with glycine powder was used. Finally fluoride gel was applied (Elmex Gelee, GABA Schweiz AG, Therwil, Switzerland) [19].

During SPC the following parameters were assessed:

- GBI [20].
- PCR [21].
- PPD.
- CAL.
- BOP.

### Statistical analysis

The individual patient was used as statistical unit. All analyses were performed on patient level. BOP was defined as the primary endpoint. All other parameters were control variables.

All teeth including 3rd molars were included into analysis.

After 10 years of SPC our group observed in periodontal compromised but treated patients a mean BOP of 18.6% [22]. We set an average periodontal BOP of 19% and expected peri-implant BOP to be 38%. To assess a difference of 19% BOP between dental implants and all teeth within the same patient for a type 1 error α < 0.05 and test power of 80% a minimal sample size of *n* = 89 is required (intra-individual comparison). In case that the difference in BOP between teeth and implants would be one to two % points smaller the actual sample size was set at *n* = 100.

A data base was generated in which the respective data were entered. The cohort under investigation was characterized with regard to patient- (age, sex, smoking, diabetes, stage, grade, number of remaining teeth, dental implants) and tooth-/implant-related criteria (frequency of PPD ≤ 3 mm [shallow pockets], 4–5 mm [moderately deep pockets], ≥ 6 mm [deep pockets]) [1, 8, 22]. Teeth and dental implants were compared with respect to BOP, GBI, PCR and frequency of PPD ≤ 3 mm, 4–5 mm, ≥ 6 mm.

Due to the fact that 16 patients were under anticoagulative medication and the putative effect of this medication on bleeding scores, the subgroups with and without anticoagulative medication were also compared.

Data were not normally distributed (Kolmogorov Smirnov test). Thus, they were expressed as medians as well as lower and upper quartiles. Comparisons between implants and teeth were made for dichotomous parameters by McNemar’s χ² and for all other parameters by Wilcoxon sign rank test. Comparisons between patients with and without anticoagulative medication were made for dichotomous parameters by χ² and for all other parameters by Mann Whitney U test.

For exploratory comparisons a logistic regression was used. The dependent variable was whether peri-implant BOP was higher than or equal to (≥) periodontal BOP (1) or not (0). The following independent variables were entered into the model: age, sex (male/female), smoking (0: non-smoker; smoker: <10 cigarettes/day; heavy smoker: ≥10 cigarettes/day); anticoagulative medication (yes; no), periodontitis stage and grade, number of teeth (including 3rd molars) and implants.

The Pearson correlation coefficient was utilized to assess the inter-individual calibration of the two examiners for the probing parameters (9 patients). The scale for interpretation was as follows: 0-0.30 indicated negligible agreement, 0.31–0.50 signified low agreement, 0.51–0.70 denoted moderate agreement, 0.71–0.90 represented high agreement and 0.91-1.0 indicated a very high agreement. Additionally, the standard deviation (SD) of individual measurements was calculated as SD = SD = SDdiff/√2, serving as an estimate for the reliability of the measurements [22].

For statistical analysis a PC program was used (Systat^™^ for Windows Version 13, Systat Inc., Evanston, USA).

## Results

One-hundred-seven patients’ charts had to be screened to include 100 patients into the study contributing a total of 2.122 teeth (including 3rd molars) and 284 implants. Seven patients were excluded due to missing HbA1c value (2), missing radiograph (2), missing PPD measurements at implants (2), missing CAL measurements (1). Females and males made up approximately half of the cohort (51/49). There were only a few smokers (6%) in the sample (Table 1). At the SPC visit considered for this study patients were under SPC for a median of 10.2 (4.5/12.9) years and had participated in a median of 17 (8.5/31) SPC visits. Ninety-seven were regular SPC compliers [18]. Sixteen patients were under anticoagulative medication (AM). Eleven patients were medicated with platelet aggregation inhibitors: acetylsalicylic acid (10), prasugrel (efient^®^) (1). One patient was medicated with acetylsalicylic acid and clopidogrel. Four patients took factor Xa inhibitors: rivaroxaban (Xarellto^®^) 2, edoxaban (Lixiana^®^) 2.

**Table 1:** Patient characteristics [number/frequency; median (lower/upper quartile)]

	*N* = 100
Age [years]	68.9 (62.6/76.5)
Female [n/%]	51/51
Number of teeth (with 3rd molars) [n]	22 (20/25)
Number of implants [n]	2 (1/4)
Non-smokers [n]	94/94
Moderate smokers (< 10 cigarettes/day) [n/%]	2/2
Heavy smokers (≥ 10 cigarettes/day) [n/%]	4/4
Well controlled diabetes (HbA1c < 7%) [n/%]	5/5
Uncontrolled diabetes (HbA1c ≥ 7%) [n/%]	1/1
Anticoagulative medication [n/%]	16/16
Localized stage III [n/%]	6/6
Generalized stage III [n/%]	57/57
Stage IV [n/%]	37/37
Grade A [n/%]	0
Grade B [n/%]	70/70
Grade C [n/%]	30/30

In 94 patients implants supported exclusively single crown or fixed dentures, in 5 exclusively removable partial dentures and in one patient both. A total of 58 patients contributed exclusively Ankylos implants (Dentsply Sirona Deutschland GmbH, Bensheim, Germany), 26 Straumann (Straumann, Basle, Switzerland)) and 7 other implant types. Four patients were treated with Straumann and other implants, four with Ankylos and Straumann implants, and one Ankylos with other implants.

Inter-individual calibration for probing parameters showed a high-positive agreement between.

PE and HP for PPD (|r| = 0.782; *P* < 0.001) and CAL-V (|r| = 0.834; *P* < 0.001; overall SD for single measurements PPD: 0.55 mm; CAL-V: 0.67 mm).

Periodontal and peri-implant conditions were predominantly healthy with median PPD of 2.3/3.0 mm, PCR of 33/16%, and GBI of 3.5/0%, respectively (Table 2). A total of 80 (28%) implants exhibited not a single site with BOP, 64 (23%) implants showed not more than one site with BOP, indicating 144 (51%) of 284 implants with peri-implant health [23]. A total of 140 implants (49%) exhibited two or more sites with BOP indicating at least peri-implant mucositis. Peri-implant BOP (24; 11.5/41.5%) was significantly higher than BOP at teeth (14; 8/21.5%) (*p* < 0.001). A median of 0 (0/1) implants exhibited no BOP and 0 (0/1) only one site with BOP. Shallow pockets (PPD 1–3 mm) were significantly more frequent in teeth (93; 87/97%) than in implants (72.5; 58/94.5%; *p* < 0.001). Moderately deep pockets (4 and 5 mm) were less frequent in teeth than implants (6; 2/11%; 22; 5.5/33%; *p* < 0.001) (Table 2).

**Table 2:** Periodontal and peri-implant variables of implants and respective teeth [median (lower/upper quartile); PPD: probing pocket depths, CAL: clinical attachment level]

Patients *N* = 100	Teeth *N* = 2.162	Implants *N* = 284	*p*
BOP/patient [%]	14 (8/21.5)	24 (11.5/41.5)	< 0.001
Plaque Control Record [%]	33 (22.5/45)	16 (0/39)	< 0.001
Gingival Bleeding Index [%]	3.5 (0.5/7)	0 (0/7.5)	0.446
PPD/patient [mm]	2.3 (2.2/2.5)	3 (2.5/3.4)	< 0.001
CAL/patient [mm]	3.1 (2.6/3.7)		
PPD < 4 mm [%]	93 (87/97)	72.5 (58/94.5)	< 0.001
PPD 4–5 mm [%]	6 (2/11)	22 (5.5/33)	< 0.001
PPD ≥ 6 mm [%]	1 (0/2)	0 (0/4)	0.081
Number of teeth/implants per patient	22 (20/25)	2 (1/4)	

Patients under AM were significantly older than no AM patients. With regard to other patient characteristics the analysis failed to find significant differences between no AM and AM (Table 3). Further, AM made no difference with regard to BOP at teeth [no AM: 14 (7.5/21.5%), AM: 14 (10/22%); *p* = 0.618] and implants [no AM: 26 (11/42%), AM: 17 (12.5/33%); *p* = 0.228] (Table 4).

**Table 3:** Patient characteristics according to anticoagulative medication [number/frequency; median (lower/upper quartile)]

Anticoagulative medication	No	Yes	
	*N* = 84	*N* = 16	*p*
Age [years]	67.9 (60.9/74.3)	77.3 (69.9/80.6)	0.001
Female [n/%]	46/54.8	5/31.3	0.085
Number of teeth (with 3rd molars) [n]	23.0 (20/25.5)	21.5 (20.5/23)	0.198
Non-smokers [n]	78/92.9	16/100	
Moderate smokers (< 10 cigarettes/day) [n/%]	2/2.4	0	0.544
Heavy smokers (≥ 10 cigarettes/day) [n/%]	4/4.8	0	
Well controlled diabetes (HbA1c < 7%) [n/%]	5/6	1/6.3	0.882
Uncontrolled diabetes (HbA1c ≥ 7%) [n/%]	1/1.2	0	
Localized stage III [n/%]	6/7.1	0	
Generalized stage III [n/%]	48/57.1	9	0.502
Stage IV [n/%]	30/11.9	7/43.8	
Grade A [n/%]	0	0	
Grade B [n/%]	56/66.7	14/87.5	0.096
Grade C [n/%]	28/33.3	2/12.5	

**Table 4:** Periodontal and peri-implant variables of implants and respective teeth per patient according to anticoagulative medication [median (lower/upper quartile); PPD: probing pocket depths, CAL: clinical attachment level, BOP: bleeding on probing]

	Teeth			Implants		
	no AM *N* = 84	AM *N* = 16	*p*	no AM *N* = 84	AM *N* = 16	*p*
BOP [%]	14 (7.5/21.5)	14 (8/21.5)	0.618	26 (11/42)	17 (12.5/33)	0.228
Full mouth plaque score [%]	31 (22/45)	42.5 (27.5/50)	0.255	16 (0/40.5)	17 (11/34)	0.488
Full mouth bleeding score [%]	3 (0/6)	4.5 (1.5/16.5)	0.161	0 (0/5.5)	0 (0/24.5)	0.237
PPD/patient [mm]	2.3 (2.2/2.5)	2.3 (2.0/2.5)	0.310	3.1 (2.6/3.4)	2.8 (2.4/3)	0.146
CAL/patient [mm]	3.1 (2.5/3.7)	3.3 (2.8/3.6)	0.554			
PPD < 4 mm [%]	92 (86.5/97)	94.5 (90.5/98)	0.192	68.5 (58/91.5)	83.5 (66.5/97.5)	0.275
PPD 4–5 mm [%]	7 (3/12)	5 (1.5/9)	0.244	25 (8.5/33)	16.5 (0/30.5)	0.169
PPD ≥ 6 mm [%]	1 (0/2)	0.5 (0/1)	0.548	0 (0/3.5)	0 (0/6.5)	0.771
Number of teeth/implants	23 (20/25.5)	21.5 (20.5/23)	0.198	2 (1/4)	2 (1/3.5)	0.919
Implants without BOP				0 (0/1)	0.5 (0/1)	0.743
Implants with one site with BOP				0 (0/1)	1 (0/1)	0.334
Implants with > one site with BOP				1 (0/2)	1 (0/1.5)	0.919

Exploratory analysis using logistic regression to identify factors influencing the dependent variable whether peri-implant BOP was higher than or equal to (≥) periodontal BOP (1) or not (0) failed to identify any factor. In 76 patients peri-implant BOP was higher than or equal to (≥) periodontal BOP.

## Discussion

Charts of SPC patients of the Center for Dentistry and Oral Medicine of Goethe-University Frankfurt were screened for patients treated with at least one dental implant until 100 patients were included in this retrospective cross-sectional study. Patients’ average age was 69 with 51 females, 6 smokers, and 16 patients under anticoagulative medication. The prevalence of peri-implant BOP was significantly higher compared to BOP at teeth.

The current study’s findings that the prevalence of peri-implant BOP, observed in a mean of 24% of sites, is significantly higher than that of mean BOP in natural teeth (14%) support the results found by Brägger et al. [24], who reported BOP at 12% of control teeth sites and a doubled incidence at implant sites. Earlier studies focusing on factors influencing peri-implant BOP found significant associations to PPD [12, 13], anterior/posterior location [13] and sex [13, 25]. However, associations of BOP to smoking, diabetes, type of implant-supported prothesis, jaw (maxilla/mandible), and implant/tooth were not found [13]. In this study mean PPD at implants are significantly higher than at teeth. However, due to the structural differences of peri-implant and periodontal tissues on average 1 mm more for peri-implant PPD is accepted [15, 26]. Additionally, to this consideration the Farina cohort consists of individuals seeking care at the Research Centre for the Study of Periodontal and Peri-implant Diseases. The cohort investigated in this study consists of periodontally compromised but treated patients under SPC. This difference may also explain the different findings. For peri-implant probing “gentle” probing aways is explicitly mentioned [2]. However, the recommended probing force (i.e., 0.2 N) [27] is very similar to the force recommended for periodontal probing (i.e., 0.25 N) [28]. Although a pressure-calibrated probe was not used in this study, both examiners, certified periodontologists with extensive clinical experience, were calibrated before the study commenced, mirroring the common clinical practice where pressure-calibrated probes are not typically used. Inter-individual calibration of probing parameters revealed a strong positive correlation between the two examiners for PPD and low SD for single measurements [22] which represents good agreement. Calibration for BOP practically cannot be performed by repeated measurements. The first measurement will have an effect on the second. Thus, calibration for BOP was not performed.

In previous scientific literature there is a broad consensus arguing that BOP is indeed a useful indicator for periodontal diagnosis with some limitations [29, 30]. However, Coli et al. (2017) question the efficacy of probing as a diagnostic tool for assessing implant health due to the histological differences in tissue structure around implants [31]. The tissue surrounding an implant is characterized by scar formation, and the connective tissue fibers are arranged differently compared to natural teeth. Furthermore, the vascularization around implants differs, which could predispose the area to increased susceptibility to bleeding, as investigated by Degidi et al. (2006). This bleeding may not necessarily be indicative of inflammation [32]. Berglundh et al. (2018) defined what constitutes peri-implant health, peri-implant mucositis, and peri-implantitis. It was established that, peri-implant mucositis is characterized by bleeding and/or suppuration on gentle probing - a criterion met by most of the implants in our study [2]. According to these definitions, most implants in the present sample (72%) would be diagnosed with peri-implant mucositis and require therapeutic intervention (i.e., removal of supramarginal biofilm). These results imply the need for further investigations as either it has to be acknowledged that implants require significantly higher therapeutic intervention compared to natural teeth or there is the need to adjust the diagnostic criteria that are applied to implants. An assessment of BOP and PPD by using the same criteria for implants as well as for teeth might lead to distorted conclusions. The high rate of at least one bleeding site per implant already led to a modification of the original definition of peri-implant health now accepting a maximum of one BOP site per implant to be in accordance with peri-implant health [23]. However, even with this less strict threshold still 49% of all implants included in this retrospective cross-sectional study fulfil the criteria of peri-implant mucositis [23]. Future research may further investigate whether the peculiarities of implants, such as a smoother surface or divergent gingival structure, are sufficiently taken into consideration during diagnostic screenings.

At the moment 3 clinical endpoints for periodontal treatment are recommended and discussed of which 2 also consider BOP. As an ideal CEP of systematic comprehensive periodontal therapy, the 2018 classification describes the state of periodontal health with the absence of clinically detectable inflammation (PPD ≤ 4 mm; no site ≥ 4 mm with bleeding on probing [BOP] and total BOP < 10%) (CEP1) [28]. The EFP S3-level clinical practice guideline defines the endpoint of therapy as no presence of PPD > 4 mm with BOP and no PPD ≥ 6 mm (CEP2) [8] and the treat-to-target approach with criteria that predict periodontal stability for the largest proportion of patients treated (≤ 4 sites with PPD ≥ 5 mm) may serve as an alternative [33]. CEP1 is quite difficult to achieve [34, 35]. In periodontally compromised but treated patients that are also treated with implants achievement of CEP 1 and 2 may be even more difficult due to the higher BOP in implants. Due to this observation, it may be considered to exclude implants when assigning CEPs. Further, in these patients the choice of intervals for SPC/supportive peri-implant care (SPIC) may be determined by peri-implant inflammation rather than periodontal inflammation [7]. Periodontal risk assessment scores (e.g., periodontal risk calculator, periodontal risk assessment) [5, 6] and the Implant Disease Risk Assessment (IDRA) [7] use the average BOP score per patient to categorized different levels of risk for periodontal/peri-implant disease. However, IDRA uses BOP averages of teeth and implants to assess risk scores. This may lead to underestimation of risk for peri-implant disease.

In SPC patients, peri-implant probing resulted in significantly higher BOP percentages than periodontal probing. Notably, the use of anticoagulative medications, including platelet aggregation inhibitors and factor Xa inhibitors, did not impact BOP rates. Comparing patients with type 1 (mild) and type 2 and 3 von Willebrand disease (VWD) with age, sex, smoking, number of teeth and periodontal disease matched controls, the bleeding disorder was also not found to affect BOP or GBI. Comparing individuals with and without type 1 VWD, GBI was significantly higher in controls (12.2%) than in VWD (10%). The study failed to find a significant difference regarding BOP between VWD (17%) and controls (17.2%). Multiple regressions identified PCR and PISA to be associated with GBI and BOP [36]. In a similar cross-sectional study comparing type 2 and 3 VWD with healthy controls, Hb1c and PCR were associated with BOP and GBI [37]. It appears that gingival/periodontal and peri-implant bleeding are more affected by inflammatory mechanisms than VWD or platelet aggregation inhibitors and factor Xa inhibitors.

Despite its implications for theory and practice, the present retrospective study has limitations. PPD were measured by two different examiners in clinical routine. Thus, they may have used different probing pressure. However, both examiners used the same type of periodontal probe that is recommended for this purpose (rigid steel probe: PCPUNC15, HuFriedy) [1]. Further, the comparison made in this study with regard to BOP was between teeth and implants within the same patient. Examiners’ differences with respect of probing pressure may have affected between patient differences (no AM versus AM). However, within patient differences (split-mouth comparison) are not affected by different examiners. The comparison between with and without AM is underpowered due to the small AM subgroup. The impossibility to calibrate the BOP parameter also is a limitation. Another limitation is limited information on implant type and abutment connection. Most implants were Ankylos and Straumann. Many patients that were evaluated in this study had their implants placed prior to periodontal treatment. Thus, we do not know when they were inserted. The time an implant is in place may have an effect on peri-implant inflammation. Future studies may prospectively compare BOP after periodontal and peri-implant probing under more strictly controlled conditions (calibrated examiner, pressure-controlled probe, type of implant). Further, the effect of AM on periodontal and peri-implant inflammation (BOP, GBI) may be investigated with equally sized test (AM) and control (nAM) groups [36, 37].

Despite the limitations of this study, we may draw the conclusions that peri-implant sites exhibit a higher prevalence of BOP compared to periodontal sites in SPC patients.

## Data Availability

The data that support the findings of this study are not available due to data protection restrictions.
